# Design and Analysis of Small Size Eddy Current Displacement Sensor

**DOI:** 10.3390/s22197444

**Published:** 2022-09-30

**Authors:** Sheng-Ching Wang, Bo-Ren Xie, San-Ming Huang

**Affiliations:** Department of Mechanical Engineering, National United University, Miaoli 360302, Taiwan

**Keywords:** eddy current displacement sensor, linear-curve fitting, temperature compensation

## Abstract

A systematic method is employed for the design and analysis of a small size eddy current (EC) displacement sensor. Simulations are first performed to determine the optimal winding structure and dimensions of the sensor. A linear-fitting approach is then developed for converting the AC displacement signal of the sensor to a DC signal. Finally, a compensation method is proposed for mitigating the temperature drift of the EC sensor under different working temperatures. The experimental results show that the proposed sensor has a sensitivity of approximately 3 μm, a working temperature range of 25–55 °C, and a linearity of ±1.025%.

## 1. Introduction

The term “eddy current” refers to the current produced in a metallic conductor by a nearby time-varying magnetic field. Eddy currents have many practical applications in industry, including position sensing, non-destructive testing, induction motors, and more [1,2]. The design and analysis of eddy current position sensors has thus attracted significant attention in the literature [3,4]. Many studies have focused on the various sources of eddy current loss in eddy current (EC) sensors and have explored the effects of unwanted losses on the sensitivity of the device [5,6]. Koibuchi et al. [7] found that the performance of EC sensors is particularly sensitive to the eddy current loss produced by the sensor casing. However, the finite element (FE) simulation results show that the casing loss could be reduced by 14.24% through appropriate shielding of the magnetic components of the sensor. Wang et al. [8] measure the normalization of non-magnetic conductors of copper (Cu), aluminum (Al), stainless steel (SS) and titanium (Ti), and show that the sensitivity of the EC sensor is mainly due to the difference in conductivity caused by different materials. The study was also showed that at a high frequency of 10 MHz, the resistance was easily affected by heat, and that it is difficult to perform the temperature compensation. Furthermore, it was shown that the sensing performance improved with both an increasing conductivity and a higher frequency.

The output signal of the EC displacement sensors should ideally vary linearly with the sensing distance. However, this is rarely the case, and most sensors exhibit a non-linear output, which varies with the material and dimensions of the target and the design and structure of the sensor. Mizuno et al. [9] examined the effects of two sensing coil materials (copper wire and magneto-plated copper wire) on the linearity range of an EC displacement sensor. The results show that the magneto-plated wire not only improved the linearity of the sensor output, but also increased the sensitivity by a factor of around 1.5 compared to that achieved using a traditional copper wire coil. Wang et al. [10] proposed a nonlinear compensation method for EC sensors based on a support vector machine (SVM), in which the setting displacement parameter was taken as the input and the voltage parameter was taken as the output.

Eddy current sensors exploit the fact that the flow of an AC current through a conducting coil generates an alternating magnetic field, which in turn induces a current flow in a nearby metallic conductor. However, when the ambient temperature rises, the resistance of the coil increases and hence the induced current flow decreases. As a result, effective methods for compensating for the thermal drift effect are required to ensure the reliability of the sensor measurements. Many temperature compensation schemes have been proposed [11,12]. Wang and Fang [13] proposed a method for reducing the thermal drift of an EC displacement sensor by two orders of magnitude by decoupling the impedance of the sensing coil into two physical quantities, namely the resistance and the inductance. The change in resistance of the coil under different temperatures was then used to calibrate the effect of the temperature on the inductance. The results obtained using a prototype device showed that the proposed approach reduced the thermal drift of the sensor to just 2.6 nm/°C. Zheng et al. [14] compensated for the temperature drift of the output signal of an EC sensor using an experimentally-derived temperature-voltage calibration equation. It was shown that the proposed method enabled the temperature drift ratio to be reduced from 28.82% to 0.9% over the temperature range of −25~80 °C.

EC displacement sensors find widespread use throughout industry nowadays [15,16,17]; ergo, the problem of developing low-cost and small-size EC displacement sensors remains an important concern. Accordingly, the present study designs, analyzes and evaluates a low-cost EC displacement sensor with dimensions of just 6 × 5 mm^2^ (diameter × height). Simulations are first performed to determine the winding structure and casing dimensions required to meet the specified sensing performance. A linear-curve fitting approach is then derived for converting the AC output signal of the sensor to a DC signal. Finally, a constant-current-based compensation method is presented for mitigating the temperature drift of the EC sensor output over a working range of 40~55 °C. The feasibility of the proposed approach is demonstrated by fabricating and characterizing an experimental prototype.

## 2. Theory of EC Displacement Sensors

**Figure 1:**
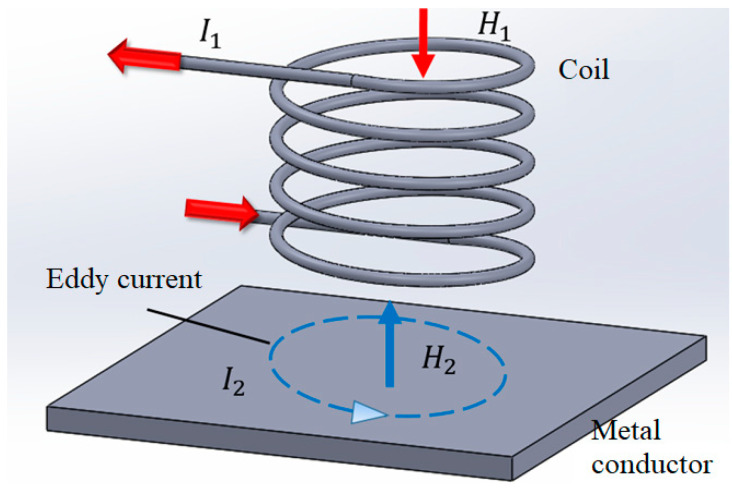
Schematic illustration of EC sensing principle.

Figure 1 presents a simple schematic illustration of the eddy current sensing process. When an alternating current, *I*_1_, is passed through the conducting coil, it generates a time-varying magnetic field, *H*_1_. If the coil lies sufficiently close to the metal conductor, a reverse magnetic field, *H*_2_, is induced in the conductor in accordance with Lenz’s law. The change in magnetic flux of this anti-coil then generates a time-varying current, *I*_2_, with a magnitude which depends on the strength of the magnetic field, the area of the coil, the rate of change of the flux, and the resistivity of the conductor.

The current distribution depth in the conductor is referred to as the skin depth and varies as a function of the resistivity *ρ* of the conductor, the magnetic permeability *μ* of the conductor, and the angular frequency *ω* of the alternating current as follows:

(1) $$\delta =\sqrt{\frac{2\rho }{\omega \mu }},$$

Eddy current sensors have different application fields depending on the design frequency of the eddy current. For a low frequency, the skin depth increases in accordance with Equation (1) and leads to a wide distribution of the eddy current in the conductor. Thus, low-frequency EC sensors are well-suited to applications such as internal defect detection, coating thickness measurement, and so on. By contrast, for a high angular frequency, the skin depth reduces, and the eddy current is distributed mainly on the surface of the conductor. Such sensors are thus best suited to measuring the feedback change between the sensor and the conductor, and are therefore mainly used for displacement, vibration, and eccentricity measurement purposes, for example.

**Figure 2:**
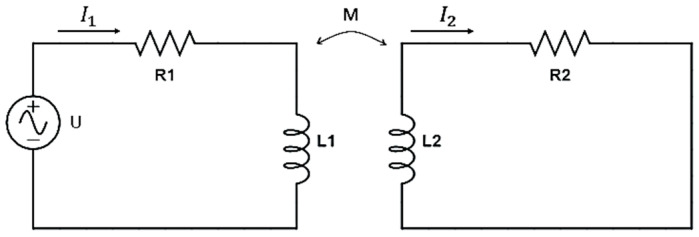
Equivalent circuit model of EC displacement sensor.

The present study focuses on the design and analysis of an EC sensor, in which the change in the eddy current feedback is used to determine the distance between the sensor head and the surface of the metal conductor. In theory, an EC displacement sensor can be represented using a mutual inductance equivalent circuit model such as that shown in Figure 2, in which the primary side corresponds to the sensing coil and the secondary side corresponds to the metal conductor. According to Kirchhoff’s second law, the total voltages around the loops in Figure 2 are given by

(2) $$R_{1}I_{1}+j\omega L_{1}I_{1}-j\omega MI_{1}=U,$$

(3) $$R_{2}I_{2}+j\omega L_{2}I_{2}-j\omega MI_{2}=0,$$

The equivalent impedance of the primary side is thus defined as

(4) $$Z=\frac{U}{I_{1}}=R_{1}+R_{2}\frac{\omega^{2}M^{2}}{R_{2}^{2}+\omega^{2}L_{2}^{2}}+j\omega [L_{1}-L_{2}\frac{\omega^{2}M^{2}}{R_{2}^{2}+\omega^{2}L_{2}^{2}}],$$

For an EC displacement sensor, the AC angular frequency, *ω*, is usually high. Consequently, Equation (3) can be simplified as

(5) $$Z=R_{1}+R_{2}\frac{M^{2}}{L_{2}^{2}}+j\omega [L_{1}-\frac{M^{2}}{L_{2}^{2}}],$$

Once the EC sensor is designed, the values of *R*_1_, *L*_1_ and *ω* are fixed. Moreover, the internal resistance *R*_2_ of the conductor is very small. Thus, the equivalent impedance of the primary side reduces to $-j\omega (M^{2}/L_{2}^{2})$. In other words, the equivalent inductance of the sensor head has a direct effect on the magnitude of the output signal. In particular, a higher inductance sensitivity leads to a greater impedance change and a higher displacement sensitivity. A change in the distance between the coil and the conductor (referred to hereafter as the sensing distance) also drives a change in the mutual inductance M.

## 3. Design and Analysis of EC Displacement Sensor

The proposed sensor was designed with an output voltage range of 1~5 V, a displacement measurement range of 0.1~0.6 mm, a sensitivity of 1 μm, a linearity of ±1%, a working temperature range of 25~55 °C, and an allowable thermal drift of ±0.8 μm/°C. Moreover, the sensor size was designed as 6 mm × 5 mm (diameter × height). It is noted that a small size design of EC sensor is the goal of this proposed study. As a result, other parameters of the proposed sensor are not comparable to the commercial one. Table 1 compares the specification of the proposed EC sensor with that of a commercial device.

The proposed sensor consisted mainly of a brass coil wound on a POM shaft and an aluminum casing. The design process commenced by performing simulations to investigate the effects of three parameters on the inductance sensitivity of the coil, namely, the inner diameter of the coil, the number of winding layers of the coil, and the inner diameter of the aluminum casing. When performing the simulation, the coil material was brass, the diameter of the brass coil was set as 0.056 mm, and an alternating voltage of 1.4 Vp-p was used as the excitation source for the coil.

**Figure 3:**
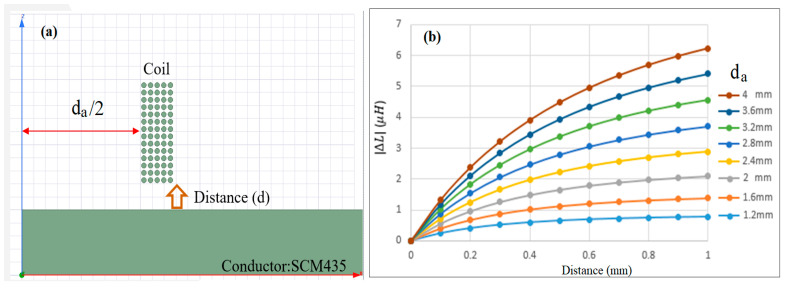
(**a**) Coil winding arrangement with 5 layers, and (**b**) effect of inner coil diameter on inductance.

Figure 3 shows the simulation results obtained for the variation of the inductance of the coil, with the sensing distance as a function of the inner diameter of the coil. The inductance is calculated by $\Delta L=N^{2}/R$, where *N* is the turns in the wire coil, and *R* is the magnetic reluctance in the magnetic circuit. The calculation is performed using Ansys Maxwell software with an input variable of the inner coil diameter. Note that the coil winding consists of 5 layers, 14 rounds, and 70 turns. As shown in Figure 3b, for each considered coil diameter, the inductance increases with an increasing distance between the sensing head and the conductor. Moreover, for a given sensing distance, the inductance increases with an increasing inner diameter of the coil.

**Figure 4:**
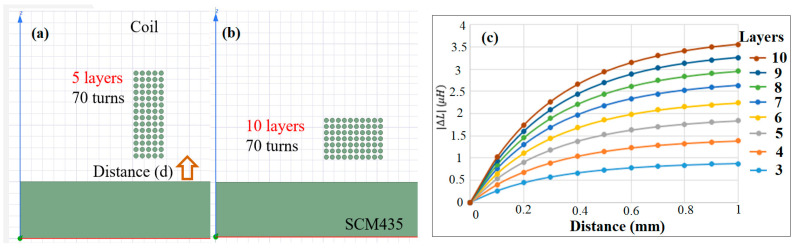
(**a**) Coil winding arrangement with 5 layers, (**b**) coil winding arrangement with 10 layers, and (**c**) effect of number of layers on inductance sensitivity.

Figure 4 shows the effect of the number of coil winding layers on the inductance sensitivity of the sensor, using Ansys Maxwell software. Figure 4a,b illustrate two typical coil arrangements with 5 layers and 10 layers, respectively. Since the total number of turns and inner coil diameter are fixed at 70 and 2 mm, respectively, the height of the coil arrangement (i.e., the number of rounds) reduces with an increasing number of layers. For example, given the use of 5 coil layers, the total number of rounds is equal to 14. However, for 10 layers, the number of rounds reduces to 7. As shown in Figure 4c, the inductance sensitivity increases with the number of layers. In other words, the inductance sensitivity improves as the coil height reduces.

**Figure 5:**
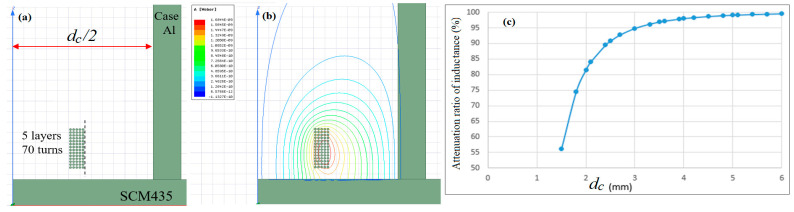
(**a**) Coil winding arrangement with 5 layers, (**b**) magnetic flux, and (**c**) effect of case inner diameter on inductance attenuation.

Figure 5 shows the magnetic flux of the coil for a winding arrangement of 5 layers and 14 rounds using Ansys Maxwell software. In performing the simulations, the casing was assumed to be fabricated of aluminum with a thickness of 0.5 mm. The metal conductor was assumed to be SCM435 alloy steel, and the sensing distance was set at 0.4 mm. As shown in Figure 5b, the equivalent inductance of the sensing coil decays more rapidly near the aluminum cover. Moreover, as shown in Figure 5c, the attenuation ratio of the inductance increases rapidly to 85% for an inner case diameter (*d_c_*) of less than 2 mm, but then increases slowly to 98% for 2 < *d_c_* < 4, and finally becomes stable at approximately 99% when *d_c_* > 4 mm. In order to keep the size of EC sensor small, the inner diameter, the inner case dimeter, and the number of layers and turns are chosen as 2 mm, 6 mm, 5, and 70, respectively, as parameters for the EC sensor prototype.

## 4. Temperature Compensation Method

**Figure 6:**
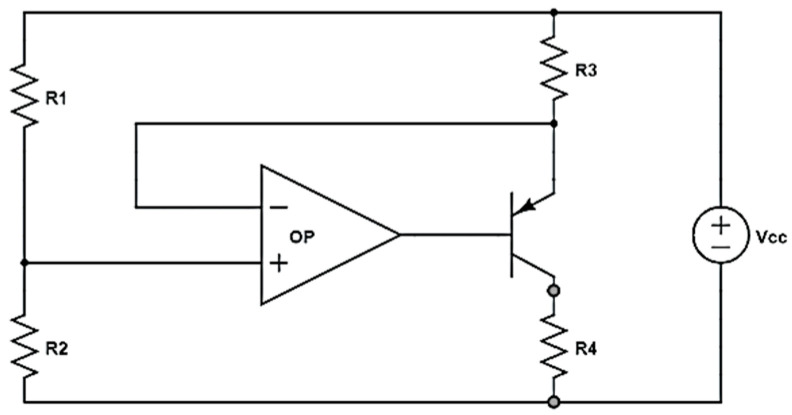
Constant-current circuit diagram.

Many studies have shown that the performance of EC displacement sensors is critically dependent on the working temperature [1,2,14,15]. Accordingly, some form of temperature compensation scheme is required such that, for a given displacement, the sensor provides the same output irrespective of the ambient temperature. In the present study, the coil is therefore modeled as a series-combined circuit of resistance and inductance, and temperature compensation is performed using the circuit design shown in Figure 6, which ensures that a constant current flows through the coil for all values of the working temperature. In Figure 6, resistance R3 serves to control the current, while resistance R4 lies on the load side and serves to load constant current. The amplifier then acts as a voltage follower to keep the *V_E_* of the PNP transistor stable. Temperature compensation is then performed by an MCU, as described in the following:

If the system satisfies $\beta R_{3}\geq 10R_{1}$, where *β* is a coefficient, a constant current *I_c_* can be obtained. Referring to Figure 6, voltage *V_1_* is defined as

(6) $$V_{1}=\frac{R_{1}V_{CC}}{R_{1}+R_{2}},$$

Voltage *V3* is then obtained from the fixed bias voltage *V_BE_* as

(7) $$V_{3}=V_{1}-V_{BE},$$

The constant current *I_C_* is then obtained as

(8) $$I_{E}=\frac{V_{3}}{R_{3}}=\left(\beta +1\right)I_{B}≅I_{C},$$

## 5. Experimental Setup and Results

**Figure 7:**
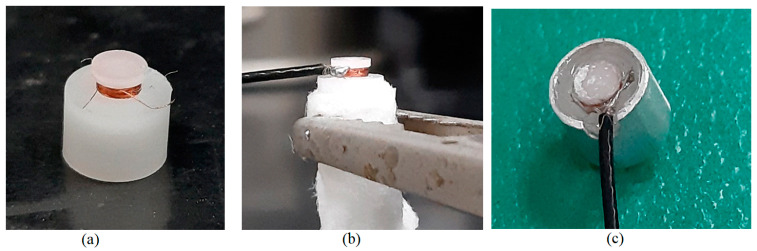
Photographs of the coil winding structure and the sensor assembly process: (**a**) coil winding, (**b**) wire connection, and (**c**) complete sensor assembly.

Figure 7 shows the coil winding structure and sensor assembly process of the prototype of the sensor head. The casing and the coil shaft were manufactured from aluminum and POM, respectively. The length of the sensor head was set at 5 mm, while the outer and inner diameters of the casing were set at 6 mm and 5 mm, respectively. The diameter and the length of the coil shaft were set at 2 mm and 0.83 mm, respectively. The coil was wound using brass wire with a diameter of 0.056 mm and consisted of 5 layers with 70 turns (Figure 7a). The output signal of the sensor was transmitted by a cable with a diameter of 0.13 mm (Figure 7b). Finally, the coil was inserted into the aluminum cover and fixed by epoxy (Figure 7c).

**Figure 8:**
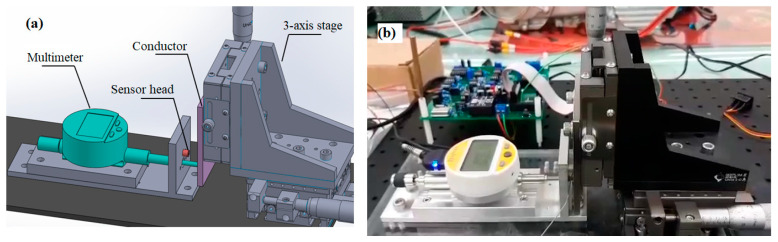
Experimental setup: (**a**) schematic illustration, and (**b**) photograph.

Figure 8 shows the experimental setup used to evaluate the performance of the prototype EC sensor. As shown in Figure 8a, the system consisted mainly of a manual three-axis stage, an SCM435 metal sample mounted on the movable stage, the prototype EC sensor mounted on a fixed stage, and a multimeter (Sylvac_NANO 805.5306) to measure the sensing distance. The EC sensor was interfaced to a circuit board (DesignSpark PCB) and a portable reconfigurable I/O (RIO) device (NI myRIO embedded device).

### Linearity Analysis Results

**Figure 9:**
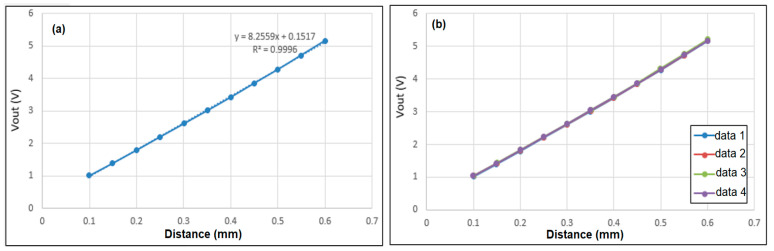
Correlation between output voltage and sensing distance over: (**a**) single test, and (**b**) four repeated tests.

Figure 9 shows the correlation between the output voltage of the EC sensor and the sensing distance under ambient temperature conditions of 25 °C. It is seen that the output voltage (y) varies linearly with the displacement (x) over the range of 0.1~0.6 mm as y = 8.2559x + 0.1517. Moreover, the correlation coefficient has a value of R^2^ = 0.9996. Thus, the linearity of the sensor is confirmed. The linearity can be evaluated as $e_{L}=(\Delta Y_{\max}/Y_{F.S})\times 100$, where $\Delta Y_{\max}$ and $Y_{F.S}$ are the maximum deviation and total range of the output voltage, respectively. Based on a detailed inspection of Figure 9a, the linearity of the sensor is found to be ±1.025%. Figure 9b shows the relationship between the output voltage and the sensing distance over four repeated trials. The repeatability of the sensor (evaluated as $e_{R}=(\Delta R_{\max}/Y_{F.S})\times 100$) is found to be 0.925% (where $\Delta R_{\max}$ = 0.037 V is the maximum deviation). Furthermore, with a minimum measured voltage of 0.024 V, the resolution of the sensor is thus determined to be 3 μm.

**Figure 10:**
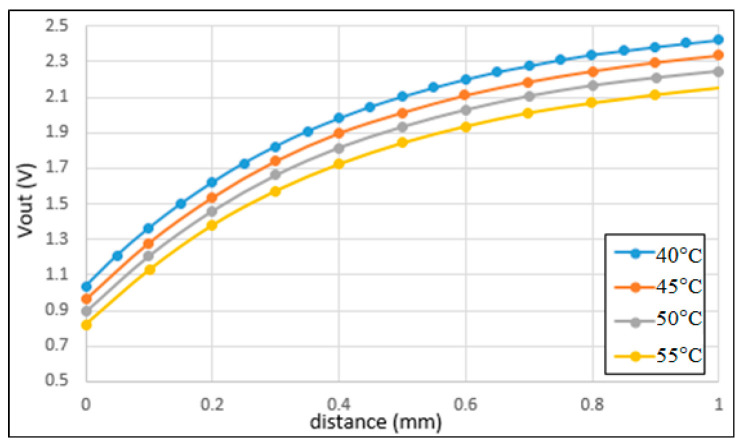
Correlation between output inductive voltage and sensing distance for working temperatures in the range of 40~55 °C.

Figure 10 shows the correlation between the output voltage and the sensing distance under different working temperatures in the range of 40~55 °C. It is noted that the temperature range was chosen based on the equipment available when performing the experiments. It is seen that both the absolute value of the output voltage and the sensitivity of the sensor reduce with an increasing temperature.

**Figure 11:**
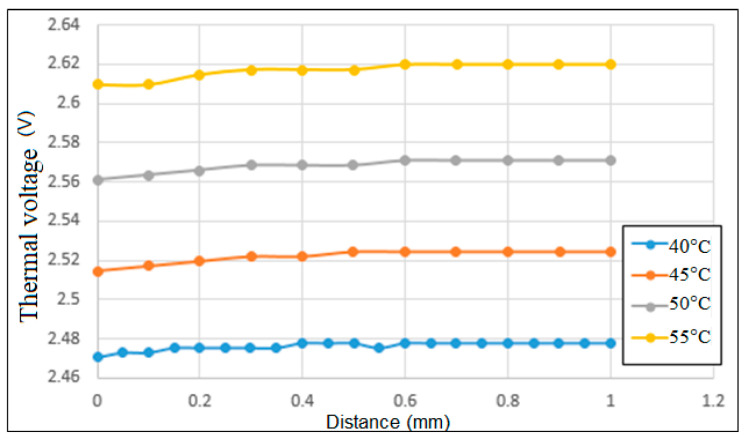
Correlation between thermal voltage and sensing distance for working temperatures in range of 40~55 °C.

In order to quantify the effect of the temperature on the output voltage, a thermal voltage parameter is introduced here, as DC voltage is a function of temperature. Figure 11 shows the variation of the thermal voltage with the sensing distance for each of the working temperatures considered in Figure 11. As shown, the thermal voltage increases from around 2.48 V to 2.62 V as the temperature increases from 40 °C to 55 °C. Moreover, for each working temperature, the thermal voltage increases slowly as the sensing distance approaches 0.4 mm, and then remains approximately constant thereafter. Overall, the results presented in Figure 11 show that the temperature drift is equal to $\delta_{vt}$ = 9 mV/°C.

**Figure 12:**
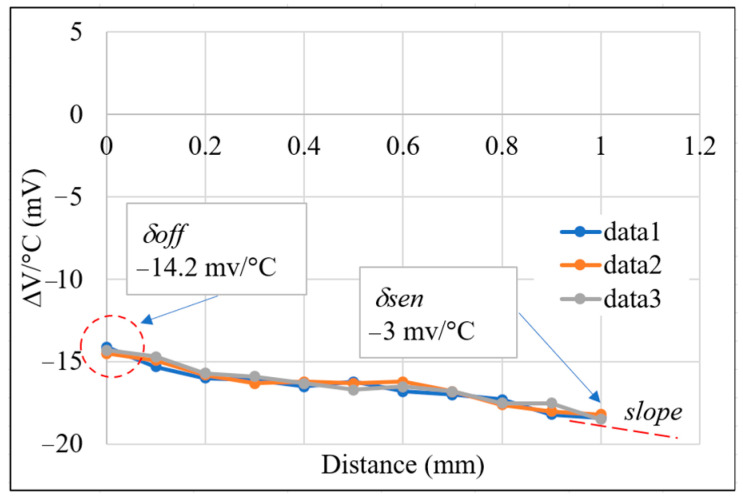
Correlation between temperature drift and sensing distance with no temperature compensation.

Figure 12 shows the correlation between the temperature drift and the sensing distance in the absence of temperature compensation. As shown, the maximum temperature drift is approximately −18.5 mV/°C, while the average variation is *δ_off_* = −14.2 mV/°C, and the sensitivity is obtained from the slope of the curves as *δ_sen_* = −3 mV/°C. It is also noted that the voltage signal obtained at a temperature of 40 °C was used for compensation purposes.

In this study, the voltage signal obtained at a temperature of 40 °C was used for compensation purposes. In particular, the temperature calibration factor was defined as

(9) $$\Delta T=(V_{t}-V_{40})\delta_{vt},$$

where *V_t_* and *V*_40_ are the measured output voltages at the working temperature *t* and the reference temperature 40 °C, respectively. In addition, $\delta_{vt}$ = 9 mV/°C is the variation of the thermal voltage obtained in Figure 11. Any measured displacement sensing signal *V* can then simply be converted to a corresponding temperature-compensated voltage *V’* as follows:

(10) $$V^{\prime }=V-\Delta T\times \delta_{off},$$

After displacement compensation, the output voltage signal obtained at any working temperature is the same at 0 mm (*V*_40°C_0m_). Therefore, the average sensitivity deviation *δ_sen_* obtained in Figure 12 can be applied to achieve the final temperature-compensated output voltage *V’’* as

(11) $$V^{\prime \prime }=V^{\prime }\times (1-\Delta T\times \delta_{sen})+V_{40{}^{\circ}C\_0mm}\times (\Delta T\times \delta_{sen}),$$

**Figure 13:**
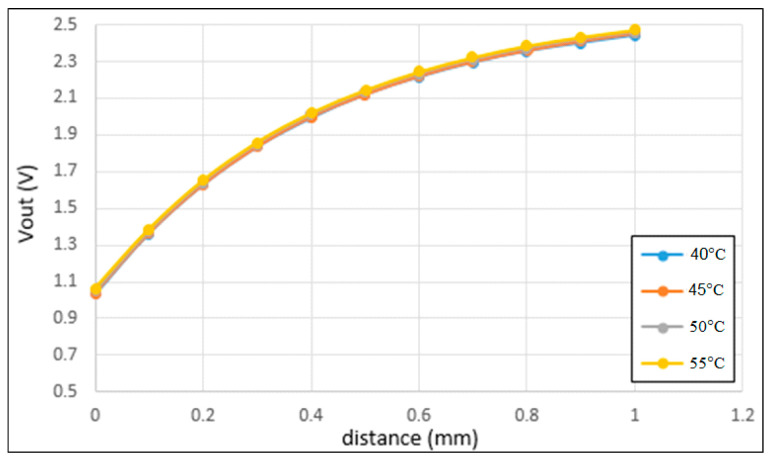
Correlation between output voltage and sensing distance following temperature compensation.

**Figure 14:**
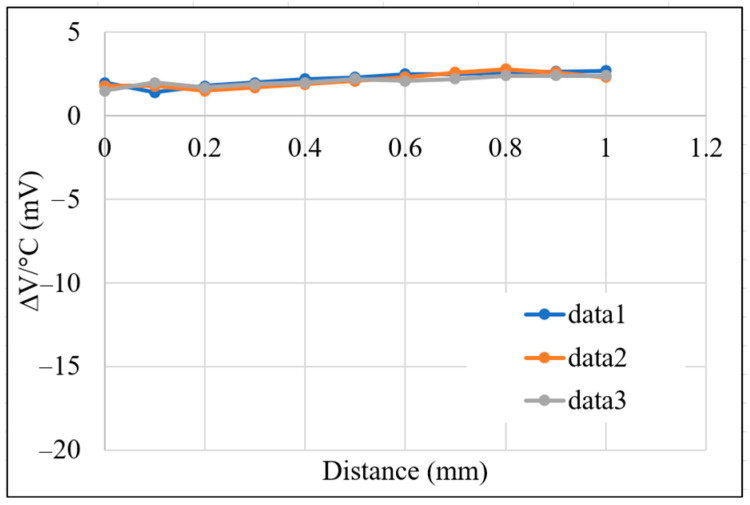
Correlation between temperature drift and sensing distance following temperature compensation.

Figure 13 shows the correlation between the temperature-compensated output voltage and the sensing distance for working temperatures in the range of 40~55 °C. As shown in Figure 13, over the sensing distance range of 0~1.0 mm, and the output voltage range of 1.05~2.45 V, the compensated temperature drift is 1.92 μm/°C (±0.96 μm/°C). Figure 14 presents the corresponding results for temperature drift with the sensing distance. As shown, the maximum temperature drift is reduced from −18.5 mV/°C with no temperature compensation (Figure 12) to just 2.7 mV/°C.

## 6. Conclusions

This study has employed a systematic approach to conducting the design and analysis of an EC displacement sensor. Finite element (FE) simulations have been performed to determine the optimal coil winding structure and sensor housing geometry. A linear-fitting approach has then been used to convert the AC output signal of the sensor to a DC signal. Finally, a temperature compensation method based on a constant-current circuit and temperature coefficient parameter has been used to minimize the temperature drift of the EC sensor and to ensure a linear and stable sensing response. The simulation results have shown that for the given sensing specification (see Table 1), the optimal coil winding structure consists of 5 layers and 70 turns. Moreover, the optimal outer diameter and thickness of the casing are 6 mm and 1 mm, respectively. The experimental results have shown that under ambient temperature conditions (25 °C), the sensor achieves a linear response over a displacement range of 0.1–0.6 mm, with a sensitivity of 3 μm and a linearity of ±1.025%. Furthermore, given the use of the proposed temperature compensation scheme, the sensor is capable of operating over a working temperature range of 40–55 °C with a temperature drift of ±0.96 μm/°C. In general, the small-size EC displacement sensor designed and fabricated in the present study provides a stable and precise measurement performance under realistic operating conditions, and thus has many potential practical applications, such as surface crack inspections, structure vibrations, etc.

## Figures and Tables

**Table 1:** Specifications of commercial AEC PU-03A EC displacement sensor and proposed sensor.

Specification	Proposed sensor	PU-03A
Measured range	0.1~0.6 mm	0~1 mm
Sensitivity	3 μm	1 μm
Linearly level	±1%	±1%
Working temperature	25~55 °C	−20~180 °C
Variable	±0.8 μm/°C	±0.8 μm/°C at −20~0 °C ±0.6 μm/°C at 0~180 °C
Dimension	ϕ6 mm × 5 mm	ϕ9 mm × 135 mm

## Data Availability

Not applicable.
